# Grassland restoration on linear landscape elements – comparing the effects of topsoil removal and topsoil transfer

**DOI:** 10.1186/s12862-024-02299-y

**Published:** 2024-08-22

**Authors:** Orsolya Valkó, András Kelemen, Orsolya Kiss, Zoltán Bátori, Réka Kiss, Balázs Deák

**Affiliations:** 1 ‘Lendület’ Seed Ecology Research Group, Institute of Ecology and Botany, HUN-REN Centre for Ecological Research, Alkotmány str. 2–4, Vácrátót, 2163 Hungary; 2 Department of Ecology, University of Szeged, Közép Fasor 52, Szeged, 6726 Hungary; 3 Institute of Animal Sciences and Wildlife Management, Faculty of Agriculture, University of Szeged, Andrássy út 15, Hódmezővásárhely, 6800 Hungary; 4 MTA-SZTE ‘Lendület’ Applied Ecology Research Group, Közép Fasor 52, Szeged, 6726 Hungary

**Keywords:** Drainage channel, Ecological restoration, Grassland recovery, Linear infrastructure, Spontaneous succession

## Abstract

Artificial linear landscape elements, including roads, pipelines, and drainage channels, are main sources of global habitat fragmentation. Restoration of natural habitats on unused linear landscape elements can increase habitat quality and connectivity without interfering with agricultural or industrial development. Despite that topsoil removal and transfer are widely applied methods in restoration projects, up to our knowledge these were previously not compared in the same study system. To address this knowledge gap, we compared spontaneous vegetation recovery after the elimination of positive (embankments) and negative landscape scars (drainage channels) in lowland alkaline landscapes in South Hungary. The novelty of our study is that we compared the fine-scale and landscape-scale results of both methods. At the fine scale, we monitored the spontaneous vegetation development on the created open surfaces in the first, second and fourth year after restoration in 160 permanent plots per year. For characterizing the habitat changes on the landscape scale, we prepared habitat maps and assigned naturalness scores to each patch before and after the restoration activities. Both restoration methods resulted in a rapid vegetation recovery at the fine scale, progressing toward the reference state. In the topsoil removal treatment, a large part of the soil seed bank was removed; therefore, the colonization of the bare surface was a slower process. Seeds of halophytes, including the endemic and protected *Suaeda pannonica*, were probably present in the deeper soil layers, and these species became established in the restored surfaces, despite being absent in the surrounding vegetation. For restoring vegetation cover, topsoil transfer was a more rapid option; however, vegetation closure and competition by generalist species and weeds hampered the establishment of target species. The removal of the landscape scars by both methods made the sites accessible for grazing. At the landscape scale, the two methods had different effects: there was a slight increase in the habitat naturalness in the topsoil removal site, and a slight decrease in the topsoil transfer site because of weed encroachment. Spreading an upper layer of nutrient-poor soil with low amounts of weed seeds, direct propagule transfer, and targeted grazing regimes could enhance restoration success.

**Supplementary Information:**

The online version contains supplementary material available at 10.1186/s12862-024-02299-y.

## Background

Linear infrastructure networks – including roads [1], pipelines [2], irrigation and drainage channels [3, 4] – are one the main sources of habitat fragmentation at a global scale. A recent analysis showed that 80% of the global terrestrial surface is fragmented into patches smaller than 1 km^2^ [5]. In the European Union, 27% of land is considered highly fragmented, where individual habitat patches are smaller than 0.02 km^2^ [6]. The adverse effects of humans on habitat connectivity occur even in protected areas: almost one-quarter of the European Natura 2000 sites are classified as highly to very-highly fragmented [7]. In fragmented landscapes, populations often have limited spatial connectivity, which has adverse effects on the population genetics as well. Limited geneflow can result in a decreased fitness of individuals and, in the worst-case scenario, can lead to local extinctions [8, 9]. Besides that, linear landscape elements often cause the fragmentation of natural habitats, they are also considered hostile environments for wildlife. For instance, the third largest human-induced mortality cause for vertebrates is human-vehicle collision [10] and at least 400 million vertebrates are roadkilled globally every year [11].

Fragmentation also has many collateral adverse effects on natural habitats. Fragmentation by linear landscape elements makes habitats more susceptible to human disturbance and habitat destruction, because such landscapes are easier to access. For instance, 95% of deforestation activities have occurred within 5.5 km distance from roads in the Amazon forests [12]. Linear landscape elements can be obstacles for natural dispersal processes and hinder habitat management [4]. Disturbances associated with the construction and maintenance of linear landscape elements can decrease the naturalness of the vegetation and facilitate the encroachment of weeds [13]. Furthermore, abandoned linear landscape elements often represent landscape scars that contribute to land degradation and erode landscape aesthetic values.

Despite the adverse ecological effects of linear landscape elements, they are integral and often inevitable components of human development, therefore strategic planning of linear infrastructure is needed for harmonizing and considering both conservation and socioeconomic factors [14]. A feasible element of strategic planning can be the restoration and rehabilitation of natural habitats on unused linear landscape elements. Such restoration and rehabilitation activities may support habitat connectivity and quality avoiding interference with agricultural or industrial development. Indeed, in certain cases, such nature conservation measures may support other sectors as well, for instance the agricultural sector, by contributing to rangeland management and providing pastures accessible for animal husbandry [4]. By eliminating linear landscape scars and restoring habitats on them, one can maximize the restoration success in a small area, and the positive effects can spill over to disproportionately larger areas [15]. Currently in the UN Decade on Ecosystem Restoration, there are several global and European-level restoration ambitions that will give new momentum for tackling habitat fragmentation [16, 17]. The EU Biodiversity Strategy 2030 aims to restore nature and reduce fragmentation of natural habitats. The recently approved EU Nature Restoration Law aims to improve the connectivity between priority habitat types, restore and rewet drained peatlands by elimination of drainage channels, and improve the connectivity between aquatic and terrestrial habitats.

In the restoration of natural habitats on linear landscape elements the first step is often some kind of topsoil movement: topsoil removal (from positive landforms standing out from the relief of the neighbouring areas, such as embankments) or topsoil transfer (to negative landforms representing a depression related to the neighbouring relief, such as channels). These measures contribute to the elimination of the physical structure of the landscape scars by levelling the soil surface. Besides soil levelling, topsoil removal is often used for eliminating the propagules of noxious plant species from degraded sites, while topsoil transfer is often used for transferring target species to sites where the seed bank is depleted [18–20]. Topsoil removal and topsoil transfer can also be used to manipulate soil nutrient levels [21–23]. Topsoil removal takes the most nutrient-rich upper soil layers away, which creates nutrient-poor conditions that can support the establishment of certain specialist target species that are poor competitors and are typical of nutrient-poor sites [23–25]. Removing topsoil from a donor site and transferring it to a receptor site is also used as a compensatory measure, e.g., when infrastructural development threatens the survival of a plant community in a donor site [18, 24].

Both topsoil removal and topsoil transfer were found to be successful methods supporting grassland restoration in several environments even without additional direct species introduction; i.e., by relying only on spontaneous recovery processes after soil transfer [24]. The success of spontaneous recovery usually depends on the initial abiotic site conditions, and on the availability of propagule sources in the soil seed bank [21]. In topsoil removal, usually the soil layers containing the highest density of the total soil seed bank are removed [22]; therefore, soil seed bank has a limited contribution to vegetation recovery. In topsoil transfer, usually subsoil layers originated from another site are spread on the receptor site, therefore the seed bank composition of the introduced soil can have a major effect on the trajectory of vegetation development at the recipient site [18, 21, 22]. In both topsoil removal and topsoil transfer, the composition of the developing plant communities is influenced also by the incoming seed rain. Since most target species characteristic of grasslands have limited spatial dispersal capacity, in degraded landscapes the seed rain often contains a high amount of weed seeds, and only a few propagules of target species [21].

As linear landscape elements usually have low surface : perimeter ratio, species from the surrounding landscape can easily colonize the restored soil surfaces [4, 26]. Site preparation and topsoil treatments before the start of spontaneous vegetation recovery are crucial, as these pre-restoration treatments can considerably affect the spatial structure and species richness of the recovering vegetation [27]. For example, [28] found that even a few centimetres difference in soil levelling can significantly influence the species composition and the diversity of the spontaneously colonizing vegetation. Despite that topsoil removal and transfer are two widely applied methods in restoration projects, up to our knowledge, the pros and cons of these two methods have not yet been compared in the same landscape.

To address the abovementioned knowledge gaps, our aim was to compare spontaneous vegetation recovery after the elimination of positive (i.e., embankments) and negative shaped landforms (i.e., channels) that acted as linear landscape scars. The novelty of our study is that we directly compared the fine-scale and landscape-scale results of both methods in the same study system. Our study system was Pannonic alkaline grasslands, which are considered priority habitats in the European Union [29]. In these habitats the adverse effects of different types of landscape scars, especially embankments and channels are widely visible, therefore restoring connectivity is crucial not only for ecological purposes, but also for increasing landscape aesthetic values, and for making the landscape accessible for livestock grazing. We compared vegetation changes after topsoil removal and topsoil transfer at two spatial scales: (i) a detailed vegetation sampling at a fine-scale and (ii) habitat mapping at the landscape-scale. We tested the following hypotheses: (i) In the topsoil removal treatment, weed encroachment is lower and vegetation development is considerably slower compared to the topsoil transfer treatment due to sparse soil seed banks. (ii) In the topsoil transfer treatment, the more abundant soil seed banks contribute to the development of a larger vegetation cover but favours weedy species. (iii) Both treatments contribute to landscape-scale improvement in habitat quality. The overall objective of our study was to evaluate whether there are trade-offs and synergies between fine-scale and landscape-scale benefits of the methods and to assess whether both options are effective at both scales.

## Materials and methods

### Study sites

The study sites are located in the Southern Tisza Valley, near the city of Szeged in the Great Hungarian Plain. The climate is continental, the mean annual temperature in the region is 10.75 ºC and the mean annual precipitation is 519 mm [30]. We studied two sites, (i) the ‘Macskási-gyepek’ site (coordinates: N 46.347540, E 20.107634), and (ii) the ‘Székalj’ site (coordinates: N 46.353968, E 20.070178). The soil of the sites belongs to the Solonetz reference soil group [31], which have a subsurface salty horizon. Both sites are characterized by alkaline grassland vegetation that is included in the Habitats Directive of the Natura 2000 system as priority habitats (*1530 Pannonic salt steppes and marshes, [29]). Before the restoration, both sites harboured grassland vegetation and linear landscape scars that were obstacles for grazing management. Before the 1960s both sites harboured undisturbed alkaline habitats. As a part of the large land transformation campaigns of the communist era, embankments were constructed on the ‘Macskási gyepek’ site and a drainage channel was established the ‘Székalj’ site in the 1960s. These landforms contributed to the degradation of the original habitats, because they modified the hydrological conditions, acted as obstacles for the grazing management, and supported the encroachment of weeds.

The ‘Macskási-gyepek’ site (~ 17 hectares) was characterised by a dense network of embankments, i.e., approx. 3–4 m wide and 1 m high positive landforms, that were considered landscape scars before the restoration. Embankments were characterised by slightly degraded dry grasslands with *Poa angustifolia*, *Bromus hordeaceus*, *Elymus repens*, and several forb species. The habitat fragments between the embankments were characterised by alkaline marshes (*Bolboschoenetum maritimi*) that composed of a mosaic with smaller stands of dry alkaline grasslands (*Achilleo setaceae*–*Festucetum pseudovinae*), open alkaline swards (*Puccinellietum distantis*), and wet meadow and marsh vegetation.

The ‘Székalj’ site (~ 62 hectares) was a typical alkaline landscape, where an approx. 3 m wide and 1 m deep drainage channel crossed the site. The site harboured several alkaline vegetation types such as open alkaline swards (*Puccinellietum distantis*, *Camphorosmetum annuae* and *Lepidietum crassifolii*), reedbeds (*Phragmitetum communis*), alkaline marshes (*Bolboschoenetum maritimi*) and alkaline wet meadows (*Agrostio stoloniferae–Alopecuretum pratensis*) were typical in the site. There were also a few dry grassland patches here (*Achilleo setaceae–Festucetum pseudovinae*, *Puccinellietum distantis*), of which some were species-poor or slightly degraded.

### Restoration treatments

In the framework of the LIFE13/NAT/HU/000081 project in March 2019 the landscape scars (i.e. embankments and channels) formerly present on the study sites were eliminated in order to restore the original open landscape and to remove obstacles and provide proper pastures for the re-introduced cattle herds. In the ‘Macskási-gyepek’ site, 790 m of embankments were eliminated by topsoil removal (hereafter called as topsoil removal treatment) and the topsoil was transferred to the ‘Székalj’ site, where the 580 m of channels were filled with the transported topsoil (hereafter called as topsoil transfer treatment). Please note that due to the nature of the restoration project, there were some limitations in our study design. The topsoil removal treatment was applied in the ‘Macskási-gyepek’ site and the topsoil transfer was applied in the ‘Székalj’ site, which is not optimal for the direct comparison of the treatments. However, the two sites were situated in very similar geographic and climatic conditions (distance between the sites was 2.8 km), and had similar vegetation types and site history (please see the previous subchapter).

**Fig. 1:**
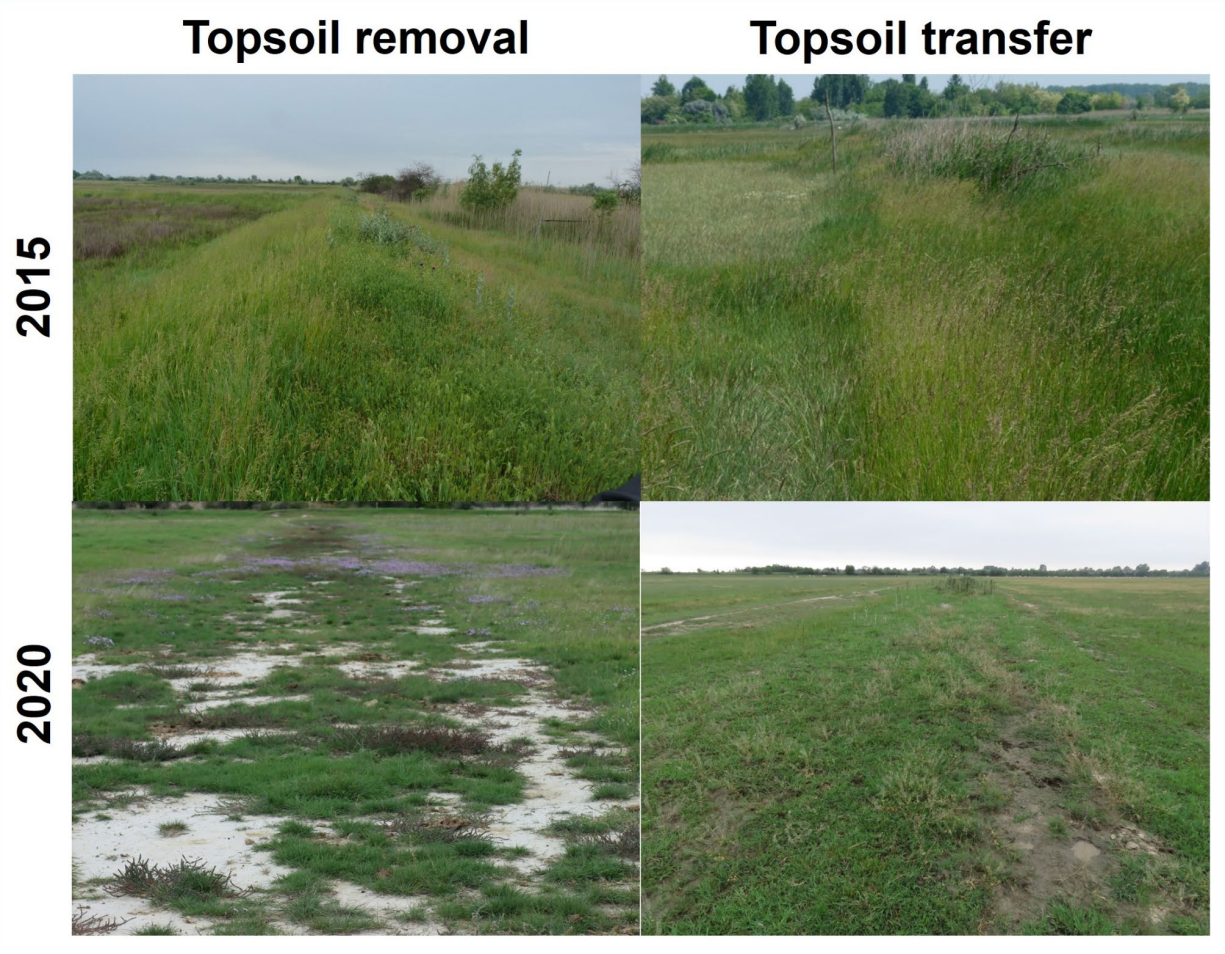
Starting conditions (2015) and post-restoration conditions (2020) in the sites restored by topsoil removal and topsoil transfer (photos by Balázs Deák and András Kelemen)

The soil surface was levelled by graders after the topsoil removal or the topsoil transfer treatments. The sites are managed by extensive Hungarian Grey cattle grazing (0.66 cattle/ha) since the summer of 2019. There were no active species introductions in the study sites, vegetation on the created open soil surfaces recovered spontaneously. The possible sources for species establishment were the soil seed bank and the seed rain from the neighbouring vegetation. The starting conditions and the post-restoration conditions are shown on Fig. 1.

### Monitoring of fine-scale vegetation dynamics

After the elimination of landscape scars by topsoil removal and topsoil transfer, we monitored the spontaneous vegetation development on the newly created open soil surfaces in September 2019, 2020, and 2022 (i.e., year 1, year 2, and year 4 after restoration). We chose this sampling period, as we expected the establishment of many halophyte species in the restored areas, which have the largest species diversity and cover in early autumn. We established 80 permanent plots of 1 m ×1 m size in both sites (i.e. 80 replicates in both the topsoil removal and topsoil transfer treatments; 160 plots in total). The plots were organized in blocks and were distributed evenly across the study sites. Four plots were placed in a block, and there was a 1 m buffer zone between the plots within each block. Plots were placed in a central position along the cross-section of the former embankments and channels to minimize edge effects. We marked the corners of all plots with underground metal sticks that enabled the precise re-survey of each plot in the consecutive years, but did not interfere with the grazing animals. We recorded the percentage cover of each vascular plant species in all plots in the three study years.

In 2022 we also sampled undisturbed alkaline grasslands that were not affected by any soil disturbance in the past centuries in both sites. For this, we designated four blocks containing in total 16 plots of 1 m ×1 m size per site (32 plots in total), situated in undisturbed vegetation patches of dry alkaline grasslands. These reference grasslands are considered the target state of the restoration.

### Survey of the total species pool and habitat mapping for monitoring of landscape-scale vegetation changes

For characterizing the initial conditions and the vegetation changes, we prepared habitat maps at both sites before (in 2015) and after (in 2020) the restoration activities by digitizing field surveys using the QGIS program [32]. To characterize the total species pool in the habitat patches surrounding the recovering soil surfaces, we recorded the list of vascular plant species in 2020. Our aim was to identify the potential propagule sources for plant establishment on the restored sites. For the evaluation of the total species pool, we designated a 200 m wide buffer zone around the restored sites. The distance threshold of 200 m was chosen as this approximates the effective dispersal distance for dry grassland species (see also [33]), so it can be assumed that species occurring within the buffer have a chance for being dispersed to and become established in the recovering vegetation.

### Data processing

Using data from the habitat maps, we calculated the area of each habitat patch in QGIS. Habitat naturalness scores ranging from 1 (completely degraded state) to 5 (natural state) were assigned to each habitat patch in both sites for 2015 and 2020 based on [34].

We classified all plant species into ecologically relevant species groups of target species and weeds. Target species were classified based on their phytosociological affiliation to classes Festuco-Puccinellietea and Thero-Salicornietea [35]. Species belonging to the categories ‘ruderal competitors’, ‘adventive competitors’, ‘adventives’ and ‘weeds’ in the Social Behavior Type (SBT) system of [35] were considered weeds. We derived ecological indicator values for nutrients (NB), water (WB), and salt (SB) for each species using the system of [35]. Then for all plots we calculated the community weighted means (CWM) of NB-, WB-, and SB-scores weighted by the cover of species to characterize the changes in the ecological requirements of the species in the recovering vegetation.

We calculated Relative Response Indices (RRI; [36]) for comparing the vegetation characteristics (i.e., total vegetation cover, cover of weeds, cover of target species, and the CWM of ecological indicator values) in the two treatments (i.e., grasslands restored by topsoil removal or topsoil transfer) and the control (i.e., local reference grasslands). This index has been used for evaluating restoration success in previous studies where local reference vegetation was also available (e.g., [37, 38]). The RRIs were calculated as follows:

RRI=(C_R_-C_T_)/(C_R_+C_T_)

where C_R_ represents a dependent variable (e.g., the cover of a functional group or cover-weighted mean ecological indicator values) in the restored plots in a given year; C_T_ represents the same dependent variable in the target grassland plots (i.e., local reference vegetation). For the calculations, we used mean values calculated for the target grassland plots per site.

Values of RRI range from − 1 to + 1. The closer the RRI is to zero, the higher the similarity of the restored vegetation to the target grasslands, whereas the closer |RRI| is to 1, the lower the similarity. We used RRIs as dependent variables in the subsequent analyses.

We used repeated measures general linear models (RM-GLMs) to evaluate vegetation changes in the three study years. Year was the within-subjects factor, and treatment (i.e., restoration method, two levels: topsoil removal or topsoil transfer) was the between-subjects factor. RRIs (calculated for the cover of functional groups and the CWMs of ecological indicator values) were used as dependent variables. All univariate statistics were calculated in SPSS 20.0.

We applied non-metric multidimensional scaling (NMDS) using Bray–Curtis dissimilarity index in CANOCO 5.0 [39] to visualize the species composition of the recovering grasslands after topsoil removal and topsoil transfer, as well as the reference grasslands. For the ordination, data was pooled at the block (four plots) level.

## Results

### Fine-scale vegetation dynamics

We recorded 116 vascular plant species in total, including 21 target species and 44 weeds in the plots during the three survey years. In the plots with topsoil removal treatment, there were 76 species in total, including 14 target species and 29 weeds. In the plots with topsoil transfer treatment, we recorded 85 species, including 14 target species and 33 weeds. Two protected species were recorded in the restored plots: *Plantago schwarzenbergiana* (with < 1% average cover in both treatments and in the reference grasslands in year 4) and *Suaeda pannonica* (with 4.9% average cover in the topsoil removal treatment in year 4 compared to 0.4% in the reference grasslands).

In year 1, no target species could become established in the restored sites. In the topsoil removal treatment, eight target species became established in year 2 and further 6 in year 4. In the topsoil transfer treatment, 13 target species became established in year 2, but 10 of them disappeared for year 4 and no new target species appeared.

We recorded 114 species in the local species pool in the Macskási-gyepek site, from which 44 became established in the plots restored by topsoil removal. We recorded 30 species in the plots restored by topsoil removal that did not occur in the local species pool. At the Székalj site, we recorded 83 species in the local species pool, from which 41 became established in the plots restored by topsoil transfer. There were 37 species in the plots restored by topsoil transfer that did not occur in the local species pool. Species that were absent in the local species pools but became established in the restored sites were mostly weeds (*Ambrosia artemisiifolia*, *Digitaria sanguinalis*, and *Echinochloa crus-gallii*), and halophyte specialist species (*Crypsis aculeata*, and *Suaeda pannonica*) at both sites.

**Fig. 2:**
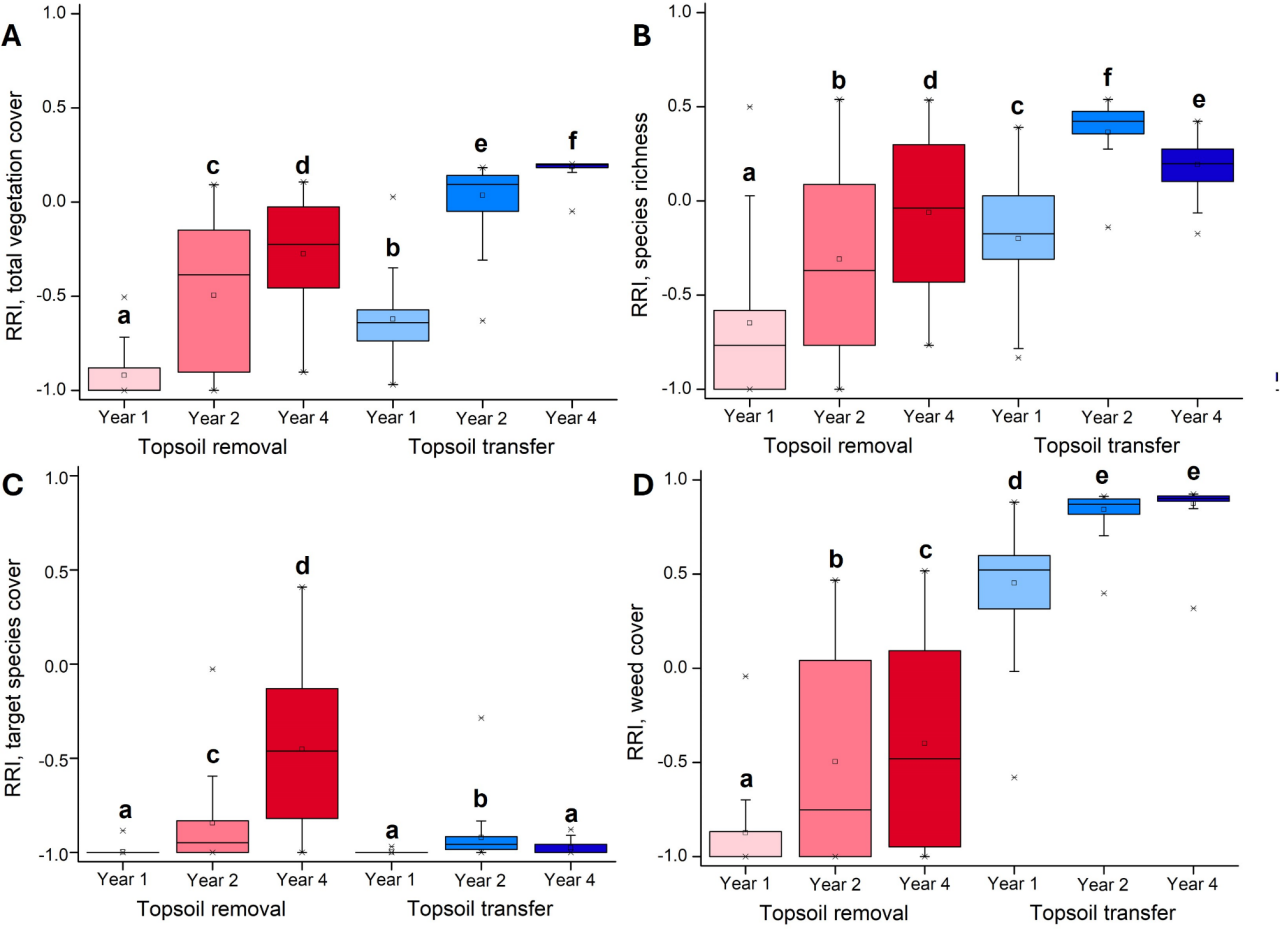
Relative response indexes (RRIs) calculated for total vegetation cover, species richness, target species cover and weed cover in the plots restored by topsoil removal (red boxes) and topsoil transfer (blue boxes) in the three studied years. Values of RRI range from − 1 to + 1. The closer the RRI is to zero, the higher the similarity of the restored vegetation to the target grasslands, whereas the closer |RRI| is to 1, the lower the similarity. Negative values indicate lower values in the restored than in the reference grasslands, and positive values indicate higher values in the restored than in the reference grasslands. Different superscript letters indicate significant differences between groups (*p* < 0.05, repeated measures general linear models and Tukey tests, *N* = 480). Boxes represent the interquartile region of value distribution (between 25% and 75% quartiles), the bold inner lines visualize the mean, vertical lines show the value range in the lower or upper quartile, respectively, and asterisks represent outliers (values that are lower or higher by 1.5× the interquartile range from the lower or upper quartile, respectively). Each boxplot represents data from 80 plots

Total vegetation cover was initially low in both treatments, but increased significantly with successional age (Appendix 1, Fig. 2A). Treatment had a significant effect on total vegetation cover, with higher scores in the topsoil transfer treatment. Vegetation cover in the topsoil transfer treatment exceeded vegetation cover in the reference grasslands for year 4. In year 1, the species richness of vegetation was lower compared to the reference, but with increasing successional age species richness increased (Appendix 1, Fig. 2B). The vegetation was more species-rich in plots restored by topsoil transfer compared to those restored by topsoil removal. The cover of target species increased significantly with successional age in the topsoil removal treatment, but not in the topsoil transfer treatment, where it remained constantly low in all years (Appendix 1, Fig. 2C). The cover of weeds increased in both treatments with successional age and was significantly higher in the topsoil transfer treatment (Appendix 1, Fig. 2D).

**Fig. 3:**
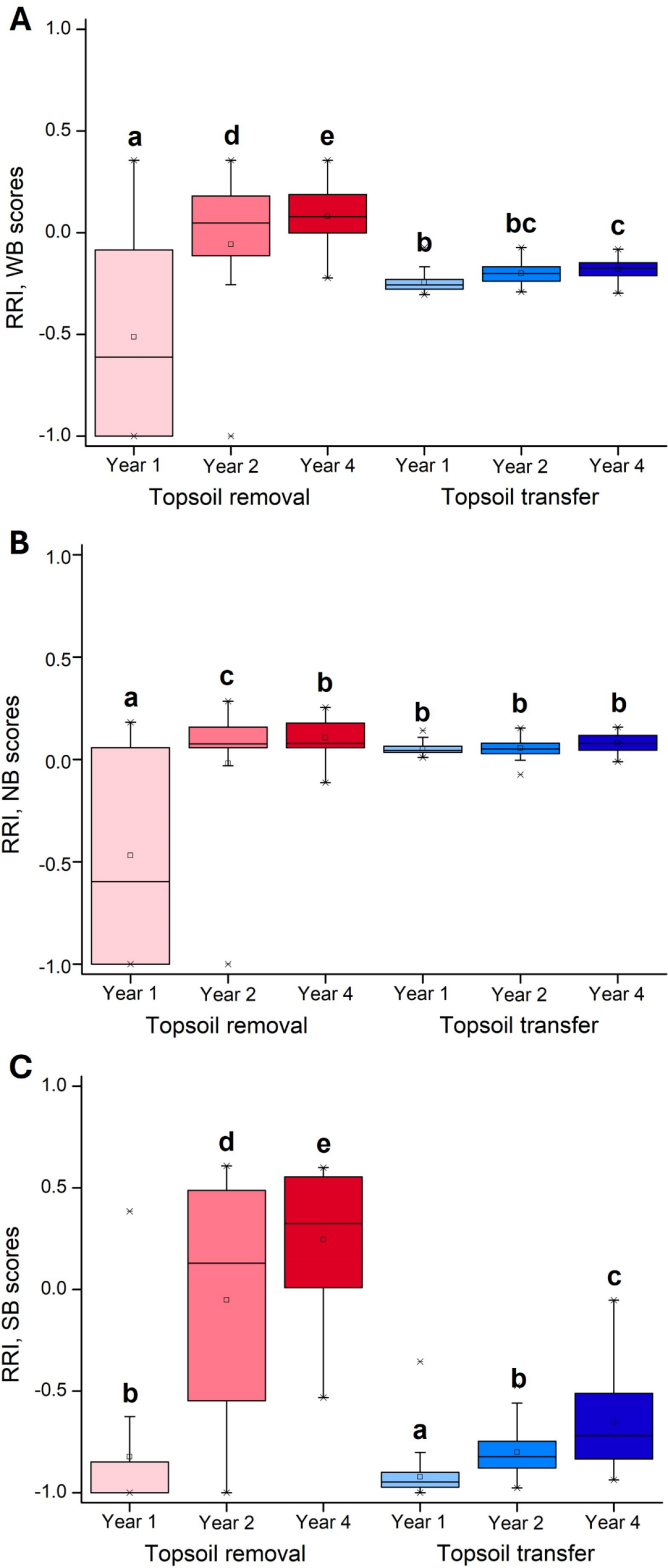
Relative response indexes (RRIs) calculated for cover-weighted mean ecological indicator values in the restored and reference plots. Notations: WB scores – cover-weighted mean ecological indicator values for water, NB scores – cover-weighted mean ecological indicator values for nutrients, SB scores – cover-weighted mean ecological indicator values for salt [35]. Values of RRI range from − 1 to + 1. The closer the RRI is to zero, the higher the similarity of the restored vegetation to the target grasslands, whereas the closer |RRI| is to 1, the lower the similarity. Negative values indicate lower values in the restored than in the reference grasslands, and positive values indicate higher values in the restored than in the reference grasslands. Different superscript letters indicate significant differences between groups (*p* < 0.05, repeated measures general linear models and Tukey tests, *N* = 480). Boxes represent the interquartile region of value distribution (between 25% and 75% quartiles), the bold inner lines visualize the mean, vertical lines show the value range in the lower or upper quartile, respectively, and asterisks represent outliers (values that are lower or higher by 1.5× the interquartile range from the lower or upper quartile, respectively). Each boxplot represents data from 80 plots

The restoration method had a significant effect on the cover-weighted mean WB-scores. The topsoil removal plots were characterized by species with higher moisture requirements than the topsoil transfer plots (Appendix 1, Fig. 3A). In year 1, the plots restored by topsoil removal were characterized by species with the lowest nutrient demand. In both treatments, the cover-weighted mean NB-scores increased with successional age and the nutrient requirements of the species at the restored plots became similar to the reference plots for year 4 (Appendix 1, Fig. 3B). Species with higher salt-tolerance (higher SB scores) were more abundant in the plots restored by topsoil removal than in those restored by topsoil transfer, and their cover increased with time in both treatments. In year 4, the restored vegetation in the topsoil removal treatment was characterized by more salt-tolerant species than the reference vegetation (Appendix 1, Fig. 3C).

**Fig. 4:**
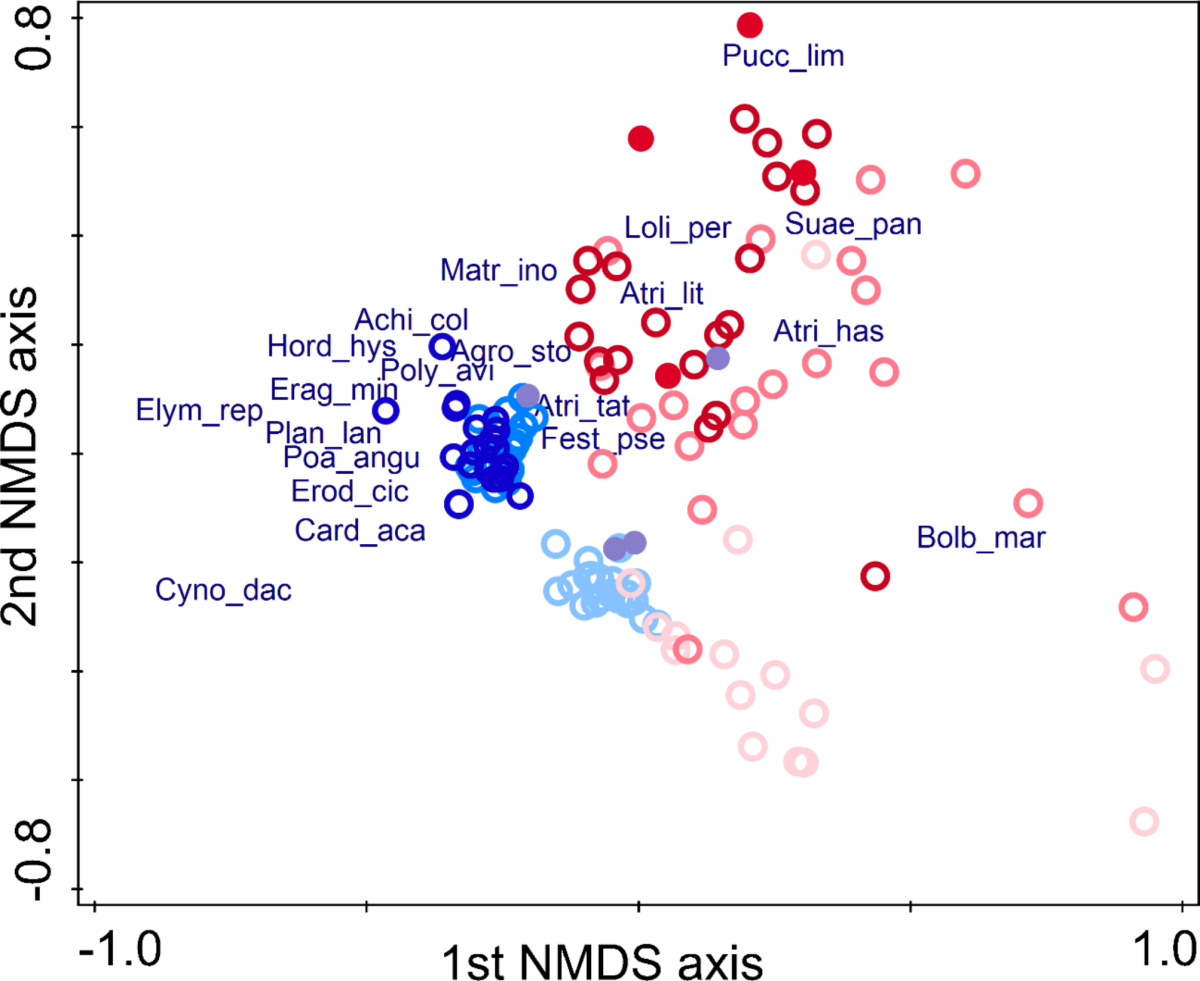
NMDS ordination showing the differences in the species composition of the plots restored by topsoil removal or topsoil transfer and the reference plots. Notations: plots with topsoil removal: – year 1 – year 2, – year 4, – reference; plots with topsoil transfer: – year 1, – year 2, – year 4, – reference. We plotted the 20 most abundant species. Species names are abbreviated using the first four letters of the genus names and first three letters of the species names. Eigenvalues were 0.49 and 0.29 for the first two axes, respectively. Cumulative explained variance of the first two axes were 49.4% and 78.32%, respectively. Each circle represents data from one block (i.e. 4 plots) in a certain year

The NMDS ordination (Fig. 4) showed a clear separation between the two treatments. The early successional vegetation (year 1) was well separated from the later years, and in both treatments the vegetation developed towards the reference state. Specialist species with high SB scores, such as *Atriplex littoralis*, *Puccinellia limosa*¸ and *Suaeda pannonica* were plotted towards the topsoil removal treatment. Most of the grazing-tolerant generalist and disturbance-tolerant species, such as *Achillea collina*, *Cynodon dactylon*, *Hordeum hystrix*, and *Plantago lanceolata*, as well as several weeds, like *Carduus acanthoides*, *Erodium cicutarium*, and *Matricaria inodora* were plotted towards the topsoil transfer treatment.

### Large-scale vegetation changes

In the site with topsoil removal (Macskási-gyepek), we distinguished 42 habitat patches in 2015 and 45 habitat patches in 2020. The average naturalness scores of the habitats increased from 3.34 (in 2015) to 3.58 (in 2020). In the site with topsoil transfer (Székalj), we distinguished 33 habitat patches in 2015 and 35 habitat patches in 2020. The average naturalness scores of the habitats decreased from 4.10 (in 2015) to 3.77 (in 2020).

## Discussion

### Effects of topsoil removal and topsoil transfer treatments on vegetation recovery

In general, both restoration methods resulted in a rapid vegetation recovery, and the vegetation development progressed toward the target state in both treatments. The total vegetation cover and species richness in both topsoil removal and topsoil transfer treatments reached the level of the target state within four years. According to the recent meta-analysis of [40], restoration methods that involve soil transfer are in general more efficient than methods using only propagule introduction without soil transfer, mainly because these methods involve the introduction of not only seeds, but also the vegetative parts of plants, and the soil microbiota.

We found that the development of vegetation cover was slower in topsoil removal treatment, which confirmed our first hypothesis. This can be partly caused by the removal of the topsoil layers with a considerable amount of the soil seed bank resulting in a slower colonisation on the bare soil surface. Probably due to the sparse vegetation and low level of competition, a vegetation characterized by pioneer specialist species, and species favouring nutrient-poor conditions became established in the first year after restoration (see also [21, 23]). With the progress of succession, the ecological niche of the species (expressed by the ecological indicator values) became increasingly similar to the target vegetation.

Topsoil removal generally removes a considerable amount of the soil seed bank (e.g. in alluvial meadows, [22]; sandy grasslands, [21]). In our study, topsoil removal affected large depths (up to 1 m), but in this study system characterized by salt-affected soils, even the deep soil layers (down to 80 cm) can contain seeds of grassland species, including several halophytes [41, 42]. We assume that seeds of halophyte species were present in the deeper soil layers (that remained intact) in the soil removal sites, because these species could establish in the site, despite they were absent in the surrounding vegetation. For instance, large populations of the endemic and protected *Suaeda pannonica* and *Plantago schwarzenbergiana*, as well as several other halophyte species, such as *Bupleurum tenuissimum* and *Crypsis aculeata*, established in the topsoil removal sites, which is a favourable process from a nature conservation viewpoint.

Besides halophytes, several weedy species that were absent in the surrounding vegetation also established in both treatments. Some of these weeds might have originated from the deeply buried seed bank, as weeds often have long-term persistent seeds that allow them to reach and remain viable in deeper soil layers [43]. Also, it is possible that some of the weed seeds were transported by the heavy machinery that was used for soil transfer and levelling. For instance, the effective spread of *Ambrosia artemisiifolia* was formerly documented on the material stuck on heavy machinery [44], and this invasive weed species occurred in our restored sites in both treatments.

For restoring vegetation cover, topsoil transfer was found to be a more rapid option compared to topsoil removal. However, for restoring target species, topsoil transfer provided insufficient results. Despite the fact that several target species established by the second year, most of them disappeared by the fourth year due to vegetation closure and competition by generalist species and weeds (see also [18]). Weed species gained increasingly high abundance in the topsoil transfer treatment, which can hinder further vegetation recovery in later years and vegetation might stuck in a weed dominated stage [45]. The topsoil transfer site received soil that likely contained large amounts of weed seeds. This is a limitation of our study that we did not have the possibility of evaluating the soil seed bank composition. Evaluating soil seed bank composition before topsoil transfer or topsoil removal can give valuable insights about the suitability of certain substrates for restoration and about the presence of propagules of target species or weeds [18, 22, 46]. Another possible explanation for the bad establishment success of the target species in the topsoil transfer treatment is that a dilution effect might occur during topsoil transfer. It means that during the transport and mixing of the soil layers, many seeds can be buried too deep to emerge [46]. In our study, the soil layers were mixed during topsoil transfer, and the original structure and functioning of the topsoil was altered. The recovery of the topsoil structure and functioning will probably require more time than the duration of our study (see [47]).

### Landscape-scale changes, and implications for restoration

The removal of the former linear landscape scars both by topsoil removal and topsoil transfer increased the landscape aesthetic values and made both sites accessible for the cattle grazing. However, the two restoration methods had different effects on the overall habitat quality at the landscape scale: we documented a slight increase in habitat naturalness at the topsoil removal site, and a slight decrease in the topsoil transfer site. A possible explanation is that on the site restored by topsoil removal (where the cover of weeds was low in the recovered vegetation), the introduced extensive grazing system increased the naturalness of the surrounding habitat patches too. Contrary, the topsoil transfer treatment resulted in a weedy vegetation and the introduced livestock could effectively disperse the weed seeds to other habitat patches by epi- [48] and endozoochory [49], resulting in a slight decrease in the conservation value of the neighbouring habitat patches. A recent meta-analysis on soil translocation experiments found that after the soil movement, the restoration success either consistently increases or decreases depending on the translocated propagule sources and on the initial site conditions [40]. This suggests that the detected landscape-level increase or decrease of habitat quality are expected to continue in the future if the management regimes at the sites remain the same.

Overall, the results of the fine-scale and landscape-scale surveys suggest that topsoil removal was an effective tool in restoring the vegetation at both spatial scales studied. In our study system, topsoil removal created new microhabitats with species composition similar to open alkaline swards and soda pans that are endangered vegetation types with high conservation value [50]. Topsoil transfer was effective for the elimination of landscape scars, but because the transferred soil originated from a degraded site, the encroachment of weeds became a problem. The idea of using the soil removed from the positive linear landscape scars to level the surface at the negative linear landscape scars can be a feasible practical solution for using similar soil texture and soil type for filling the gaps. This can also minimize disturbance of the donor site, which can be problematic in topsoil transfer actions [51]. However, based on our results, we recommend that other restoration measures should be applied to avoid the encroachment of weeds at the receptor site, such as spreading an upper layer of nutrient-poor soil containing low amount of weed seeds. For supporting the establishment of target species, hay transfer or other type of propagule transfer could be combined with topsoil transfer [38, 40]. Using targeted grazing regimes [52], e.g. by moving the animals from the habitat patches with higher naturalness values towards those with lower naturalness values can support the recovery of the habitats in the long run.

### Electronic supplementary material

Below is the link to the electronic supplementary material.

Supplementary Material 1

## Data Availability

The datasets analysed during the current study are available from the corresponding author on reasonable request.
