# Supplementary material for: Grassland restoration on linear landscape elements – comparing the effects of topsoil removal and topsoil transfer
Source: BMC Ecol Evol. 2024 Aug 22;24:112. doi: 10.1186/s12862-024-02299-y (PMC11340085; doi:10.1186/s12862-024-02299-y)
Supplement: Supplementary file 1 — Supplementary Material 1 [file 12862_2024_2299_MOESM1_ESM.docx]

**Appendix 1.** Results of the RM-GLM models on the effect of restoration method (two levels: topsoil removal, topsoil transfer), year (three levels) and their interaction on relative response indexes (RRIs) calculated for the studied vegetation characteristics. Significant effects are marked with boldface.

|  | **Restoration method** | | **Year** | | **Restoration method × year** | | **Block (random effect)** | |
| --- | --- | --- | --- | --- | --- | --- | --- | --- |
| **Vegetation characteristics** | **F** | ***p*** | **F** | ***p*** | **F** | ***p*** | **F** | ***p*** |
| RRI, Total cover | **40.761** | **<0.001** | **165.495** | **<0.001** | 0.089 | 0.766 | **5.832** | **0.017** |
| RRI, Species richness | **64.113** | **<0.001** | **76.053** | **<0.001** | **52.580** | **<0.001** | **10.605** | **0.001** |
| RRI, Target species cover | **26.180** | **<0.001** | **9.157** | **0.003** | **31.870** | **<0.001** | 0.023 | 0.878 |
| RRI, Weed cover | **162.082** | **<0.001** | 66.906 | **<0.001** | **16.157** | **<0.001** | **14.509** | **<0.001** |
| RRI, NB-score | 0.757 | 0.386 | **3.870** | **0.050** | 0.160 | 0.690 | **34.033** | **<0.001** |
| RRI, WB-score | **36.206** | **<0.001** | 2.560 | 0.112 | 0.066 | 0.797 | **33.373** | **<0.001** |
| RRI, SB-score | **105.786** | **<0.001** | **7.061** | **0.009** | **13.146** | **<0.001** | 0.223 | 0.637 |
